# Methyl Jasmonate Enhances Saponin Accumulation in Cultured *Panax notoginseng* Adventitious Roots

**DOI:** 10.3390/plants14223462

**Published:** 2025-11-13

**Authors:** Kaiyang Liu, Ping Li, Wenlan Li

**Affiliations:** State Key Laboratory for Conservation and Utilization of Subtropical Agro-Bioresources, College of Life Science and Technology, Guangxi University, Nanning 530004, China; 2408301045@st.gxu.edu.cn (K.L.); liping_24@126.com (P.L.)

**Keywords:** *Panax notoginseng*, notoginsenosides, methyl jasmonate, gene expression, LC-MS, untargeted metabolomics

## Abstract

*Panax notoginseng* is a valuable medicinal herb, but its sustainable production is constrained by long cultivation cycles and continuous cropping obstacles. Adventitious root culture presents a viable alternative. This study establishes a robust and sustainable platform for the efficient production of ginsenosides in *P. notoginseng* adventitious root cultures. We first systematically optimized the culture system, identifying leaf segments as the optimal explant due to their high callus induction rate (95.77%), low contamination, and renewable nature. Combined with optimized bioprocess parameters (3 g/L inoculation density, 50 g/L sucrose), this strategy addressed key practical bottlenecks. Beyond methodological advancement, our research provides novel mechanistic insights into the action of methyl jasmonate (MeJA). A key finding is the discovery that MeJA functions as a ‘precision metabolic switch,’ differentially regulating a critical branch point in the saponin pathway. It coordinately upregulates the protopanaxadiol (PPD)-type gene *CYP716A47* while downregulating the protopanaxatriol (PPT)-type gene *CYP716A53v2* that genetically explains the directed enrichment of specific ginsenosides. This integrated approach not only advances the fundamental understanding of elicitor action but also provides a scalable and controllable system for the industrial production of *P. notoginseng* phytopharmaceuticals with tailored saponin profiles.

## 1. Introduction

*Panax notoginseng* (Burk.) F.H. Chen is a perennial herbaceous plant belonging to the Araliaceae family and the *Panax* genus. It is primarily distributed in Yunnan, Guangxi, and Sichuan provinces in China. *P. notoginseng* is a traditional Chinese medicinal herb with a history of over 600 years of use and 400 years of cultivation [1]. It contains various bioactive compounds, including triterpenoid saponins, flavonoids, polysaccharides, and amino acids. Among these, dammarane-type tetracyclic triterpenoid saponins are the main active components of *Panax* plants, and over 270 types of ginsenosides and notoginsenosides have been isolated from different parts of *P. notoginseng* [2]. Currently, more than 400 traditional Chinese medicines contain *P. notoginseng* as a key ingredient, including Yunnan Baiyao, Compound Danshen Dripping Pills, and Pianzihuang. In 2019, the annual demand for *Panax notoginseng* was approximately 8000 tons at that time, serving as a historical benchmark for its market need [3]. Modern pharmacological studies have shown that ginsenosides possess significant medicinal value, with notable effects in anti-cancer [4], antioxidant, anti-inflammatory [5], cardiovascular disease prevention [6], immune enhancement, and neurological disease treatment.

The long cultivation cycle and severe replant problem of *P. notoginseng* have led to a shortage of resources [7]. Soil Sickness result from the dysbiosis of the plant–soil–microbe holobiont system. The primary causative factors include: a deleterious shift in the rhizosphere microbial community structure, the accumulation of allelopathic or autotoxic compounds, and the degradation of soil physicochemical properties. Obtaining active secondary metabolites such as triterpenoid saponins through plant cell metabolic pathways is an effective way to meet market demand. Compared to cell suspension culture, adventitious root culture offers higher efficiency and stability in secondary metabolite synthesis. It is known that optimizing the culture medium composition and conditions can enhance the synthesis and accumulation of total saponins in *P. notoginseng*. Additionally, the use of elicitors is a common strategy to promote the accumulation of secondary metabolites. Studies have shown that methyl jasmonate (MeJA) significantly affects the accumulation of secondary metabolites in various plant cell and organ cultures, such as flavonoids [8], alkaloids [9], phenolic acids (e.g., chlorogenic acid [10]), and terpenoids (e.g., paclitaxel [11]). MeJA is also widely used in *Panax ginseng*, effectively promoting the accumulation of ginsenosides [12]. However, there is still a significant gap compared to natural roots [13], and the variety of saponin monomers in cultured roots is often limited, with the absence of major saponin monomers [12]. How to obtain a rich variety of saponin monomers and high yields of triterpenoid saponins from *P. notoginseng* suspension cultures remains a challenge. A more detailed understanding of how MeJA influences the biosynthetic pathway—particularly its effect on the expression of genes responsible for the divergence between protopanaxadiol (PPD)-type and protopanaxatriol (PPT)-type saponins—is needed to address this limitation. Therefore, this study had two interconnected objectives: (1) to establish a robust adventitious root culture system by systematically selecting optimal explants and culture conditions, and (2) to investigate the molecular-level effects of MeJA by analyzing its impact on the expression of key saponin biosynthesis genes, thereby providing insights for better pathway control.

LC-MS visualizes ion flow intensity as peak areas, which are linearly related to metabolite concentrations, providing relevant biological information through differential analysis [14]. LC-MS-based metabolomics is an effective method for comprehensively evaluating the quality of medicinal plants [15]. By detecting the composition and content of metabolites, it can reflect changes in metabolites after induction or interference.

This study aims to establish and optimize an adventitious root suspension culture system for *P. notoginseng* and apply MeJA to promote the accumulation of total saponins in adventitious roots. The study will analyze the induction effect of MeJA on adventitious roots from a metabolic perspective, combined with changes in physiological and biochemical indicators and the expression of ginsenoside biosynthesis genes, to explore the impact of MeJA on metabolite synthesis and its induction mechanism, thereby advancing research on *P. notoginseng* adventitious root suspension culture.

## 2. Results and Discussion

### 2.1. Effect of Different Explants and Plant Hormones on Callus Induction

**Figure 1:**
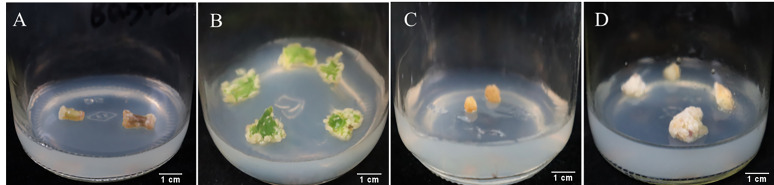
Growth of callus tissues from different explants. (**A**) is the expansion of the two ends of the stem incision and then the callus is generated. (**B**) is the callus growth induced by leaves for 30 days. (**C**) is the callus growth of root section for 50 days. (**D**) is the proliferation of callus after 30 days of subculture.

As shown in Supplementary Table S1, the three explants showed high callus induction rates under various hormone combinations. When roots were used as explants, the overall induction rate was lower compared to stems and leaves, with half of the hormone combinations failing to induce callus growth. The highest callus induction rate (86.81%) was observed in medium 4 (MS + 6-BA 1.0 mg/L + 2,4-D 1.0 mg/L), but the initiation time was slow, and callus growth was also slow, with only a small amount of callus growth observed after 50 days (Figure 1C). When stems were used as explants, dedifferentiation occurred the fastest, with swelling observed at both ends of the stem incision after 7~10 days (Figure 1A). Medium 1 (MS + 6-BA 1.0 mg/L + 2,4-D 1.5 mg/L + KT 0.5 mg/L) showed the highest callus induction rate (92.83%), significantly different from other media. When leaves were used as explants, medium 11 (MS + 6-BA 1.5 mg/L + 2,4-D 1.0 mg/L) showed the highest callus induction rate (95.77%) after 30 days. During the culture process, the edges of the leaves slightly bulged and irregularly curved inward, followed by the formation of compact, light green callus at the incision sites and along the minor veins (Figure 1B). Based on these results, leaves and stems were considered the best explants. Due to the abundance of leaves, ease of collection, and low contamination rate in preliminary experiments, leaves were deemed the most suitable explant for callus induction in *P. notoginseng*.

The selection of an optimal explant is a critical first step in establishing an efficient in vitro culture system, and our results demonstrate a clear trade-off among different explants of *P. notoginseng* when considering induction efficiency, practical availability, and procedural sterility.

While stem segments exhibited the most rapid initiation of callus formation, their practical application is significantly limited by two major constraints. Firstly, the high contamination rate associated with stem explants, likely due to their complex internal structure and higher density of potential microbial niches, poses a significant challenge for obtaining sterile cultures. Secondly, and perhaps more critically for perennial medicinal plants, the stem is a singular and vital organ of the mother plant. Its removal for explant purposes typically results in the death of the donor plant, making this approach unsustainable for large-scale or continuous culture establishment. This limitation is seldom discussed but is of paramount importance for conservation and commercial propagation.

Conversely, root explants, while theoretically abundant, present their own set of challenges. The intricate surface and endogenous microflora of roots necessitate prolonged and harsh sterilization protocols, often involving high concentrations of disinfectants like HgCl_2_. As documented in *P. notoginseng* by Gao et al. [16], such aggressive sterilization can be phytotoxic, damaging explant tissues and ultimately leading to a marked decrease in callus induction rates and vitality, which aligns with our observations of the lowest overall induction rates in root explants.

In this context, leaf explants emerge as the most balanced and advantageous choice. As confirmed in our study and supported by previous work [13], leaves consistently yield high callus induction rates. The hormonal combination identified in our study (MS medium supplemented with 6-BA 1.5 mg/L and 2,4-D 1.0 mg/L) proved highly effective for leaf explants, achieving an induction rate of 95.77%. This efficacy can be attributed to the optimal balance between the cytokinin (6-BA) that promotes cell division and the auxin (2,4-D) that induces dedifferentiation, a synergy that is well-established for inducing callus from leaf tissues in *Panax* species. From a practical standpoint, leaves offer a renewable resource from the mother plant without causing fatal damage, have a relatively smooth surface that is easier to sterilize effectively, and consequently exhibited the lowest contamination rate in our preliminary experiments. Therefore, considering the synergistic benefits of high induction efficiency, low contamination risk, and sustainable sourcing, leaf explants are unequivocally recommended as the most suitable and reliable explant for the efficient induction of callus in *P. notoginseng*.

### 2.2. Adventitious Root Differentiation

**Figure 2:**
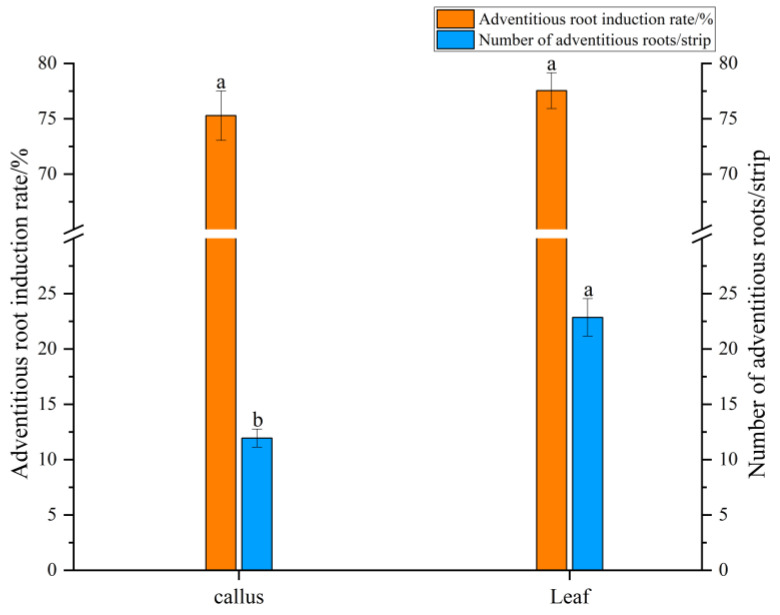
Differentiation of adventitious roots from callus and leaf explants. Data were collected on day 30 for callus and day 50 for leaf explants. Different lower case between explants indicates *p* < 0.05. Error bars represent the standard error of each mean (n = 3).

**Figure 3:**
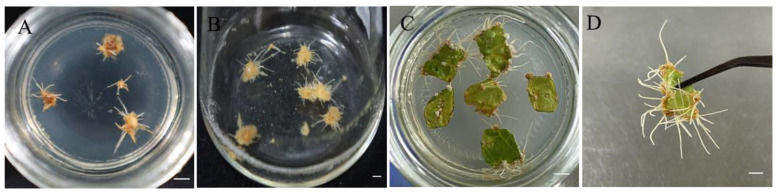
Differentiation of *Panax notoginseng* adventitious roots. (**A**) Differentiation of adventitious roots in callus tissue; (**B**) Adventitious root suspension culture; (**C**,**D**) Leaf differentiation adventitious roots. Bars = 1 cm.

As shown in Figure 2, when callus was used as the explant, the adventitious root differentiation rate was 75.28%, with an average of 11.94 roots per callus, showing good growth (Figure 3A). After appropriate segmentation and transfer to liquid medium, the adventitious roots grew rapidly and appeared vibrant after 20 days of suspension culture (Figure 3B). When sterile leaves were inoculated into the differentiation medium, a small amount of callus formed around the leaf incision and along the minor veins after 30 days, from which adventitious roots gradually differentiated. After 50 days, the differentiation rate reached 77.54%, with an average of 22.85 adventitious roots per leaf (Figure 3C,D). Compared to callus inoculation, direct leaf inoculation resulted in more stable adventitious root differentiation, with a higher number of roots and significant differences compared to callus inoculation.

Our results clearly demonstrated that leaf explants were superior to callus for adventitious root differentiation. This advantage stems from fundamental differences in their biological nature. Leaf explants possess pre-existing meristematic tissues and organized vascular structures that can be directly reprogrammed into root primordia under appropriate hormonal induction [17]. In contrast, callus represents a dedifferentiated cell mass that must undergo complete cellular reprogramming to establish new developmental pathways, a process that is inherently more variable and less efficient [18]. The auxin combination of IBA and NAA in our culture medium effectively activated the innate organogenic potential present in leaf tissues, leading to more reliable and prolific root formation compared to the indirect pathway through callus.

### 2.3. Effect of Inoculation Amount and Sucrose Concentration on Biomass and Total Saponin Content

**Figure 4:**
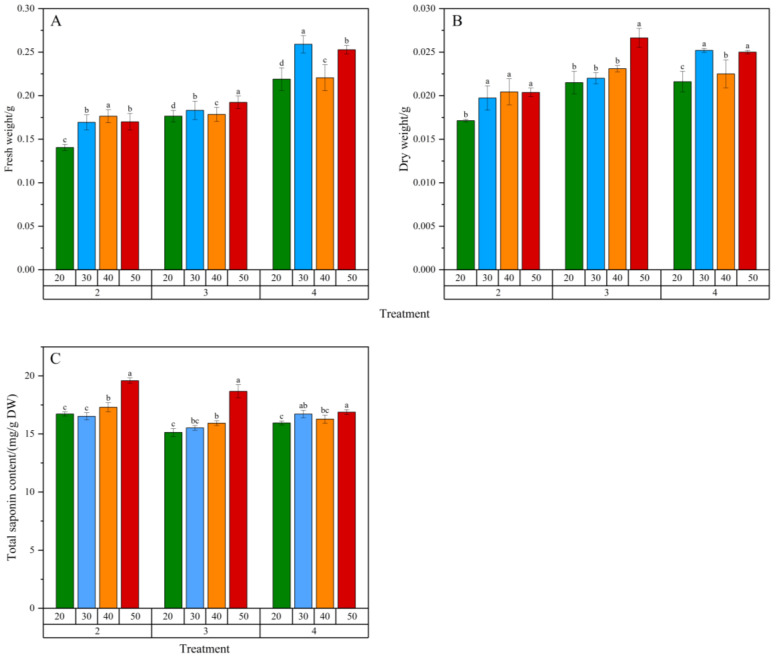
Effects of inoculation amount and sucrose concentration in culture medium on the biomass and total saponin content of *Panax notoginseng* adventitious roots. (**A**–**C**) represent fresh weight (FW), dry weight (DW) and total saponin content of *Panax notoginseng* adventitious roots, respectively. Net increase in fresh weight (FW) and dry weight (DW) of adventitious roots, representing the biomass harvested from a 50 mL culture volume relative to the initial inoculation weight. Horizontal coordinates 20, 30, 40, 50 represent sucrose concentration (g/L) in the medium; The horizontal coordinates 2, 3 and 4 represent the inoculation amount of adventitious roots (g/L); Different lowercase letters in the same dose indicate *p* < 0.05. Error bars represent the standard error of each mean (n = 3).

As shown in Figure 4, different inoculation amounts and sucrose concentrations significantly affected the biomass accumulation and total saponin content of adventitious roots. When the inoculation amount was 2 g/L or 3 g/L, the biomass of adventitious roots generally increased with higher sucrose concentrations, with the highest dry weight accumulation observed at 3 g/L inoculation and 50 g/L sucrose. At 4 g/L inoculation and 30 g/L sucrose, the biomass of adventitious roots was the highest, significantly different from other sucrose concentrations. The highest total saponin content was observed at 50 g/L sucrose for all inoculation amounts (2~4 g/L), consistent with the results of Zhao et al. [13]. From the perspective of biomass and total saponin content, sucrose concentration positively correlated with adventitious root growth and secondary metabolite production, promoting biomass accumulation and increasing total saponin content.

At 2 g/L inoculation and 50 g/L sucrose, the total saponin content in adventitious roots was the highest, at 19.6 mg/g DW. Considering economic efficiency and cultivation cycle, 3 g/L inoculation and 50 g/L sucrose were optimal, resulting in the highest dry weight accumulation and total saponin yield over a 30-day cultivation period.

The optimization of inoculation density and carbon source concentration is pivotal for balancing biomass accumulation and secondary metabolite production in plant in vitro cultures. Our results demonstrate a complex interplay between these two factors in *P. notoginseng* adventitious root cultures.

The positive correlation between sucrose concentration (20–50 g/L) and both biomass and total saponin accumulation underscores the dual role of sucrose as both a carbon skeleton for growth and an energy source for secondary metabolism. Higher sucrose levels (50 g/L) likely created an elevated carbon-to-nitrogen ratio and osmotic pressure, which are known elicitation signals that divert metabolic flux from primary growth towards the synthesis of defensive secondary compounds like saponins [19]. This explains the highest total saponin content observed at 50 g/L sucrose across all inoculation densities.

Regarding inoculation density, the highest biomass accumulation at 3 g/L, as opposed to 4 g/L, can be attributed to the balance between resource availability and growth-inhibiting factors. At a lower density (2 g/L), the culture may have initially underutilized the available nutrients and space, resulting in lower final biomass. At a higher density (4 g/L), the roots likely entered a phase of early resource competition and accumulated inhibitory levels of phenolic compounds or other waste metabolites in the medium, leading to growth suppression [20]. The 3 g/L density appears to represent the optimal equilibrium, allowing for efficient nutrient uptake and ample growth space without significant mutual shading or inhibitor accumulation.

The decision to select 3 g/L with 50 g/L sucrose as the optimal condition, despite 2 g/L yielding a slightly higher content of saponins, is a classic and pragmatic trade-off in bioprocess engineering. This combination prioritized the highest total yield of saponins per culture vessel by maximizing the product of biomass and content, which is the most critical parameter for scaling up production [21]. Therefore, our optimized conditions successfully harness the physiological principles of nutrient allocation and elicitation to maximize the economic output of the *P. notoginseng* adventitious root culture system.

### 2.4. Effect of Methyl Jasmonate on Biomass and Total Saponin Content

**Figure 5:**
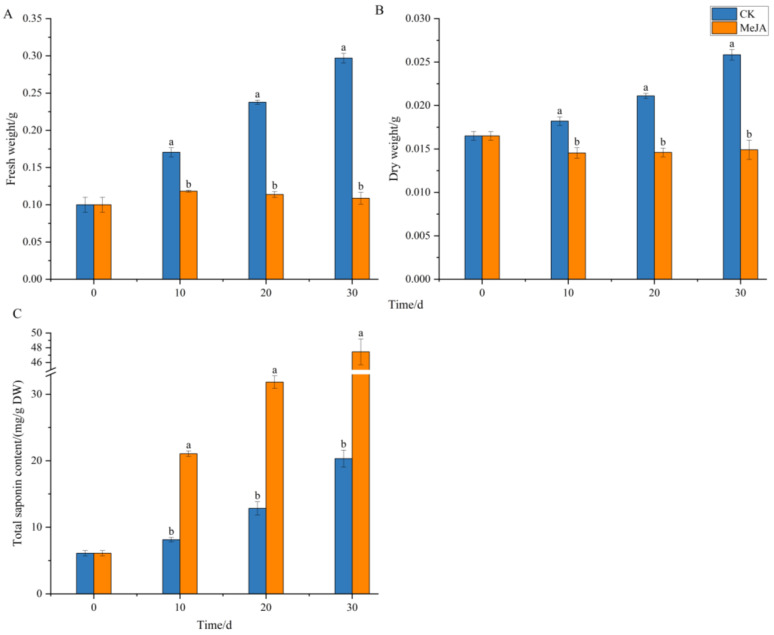
Effects of methyl jasmonate on the biomass and total saponin content of *Panax notoginseng* adventitious roots. (**A**–**C**) represent fresh weight(FW), dry weight(DW) and total saponin content of *Panax notoginseng* adventitious root, respectively. CK refers to the control group, and MeJA represents the treatment with 200 µM Methyl Jasmonate. Different lowercase letters at the same cultivation time indicate *p* < 0.05. Error bars represent the standard error of each mean (n = 3).

As shown in Figure 5, the biomass of adventitious roots in the control group (CK) increased continuously over the 30-day cultivation period. The total saponin content increased slowly from 0~10 days, likely due to the adaptation of adventitious roots to the culture environment and the abundance of nutrients in the medium, resulting in rapid fresh weight growth. From 10~30 days, as the biomass increased, the total saponin content also increased significantly. In the MeJA-treated group, the fresh weight of adventitious roots increased slightly initially but did not increase further, while the dry weight did not show significant growth over the cultivation period. However, the total saponin content increased continuously from 0~30 days, significantly higher than the control group, reaching 47.44 mg/g DW at 30 days, 2.34 times that of the control group (20.32 mg/g DW).

The observed trade-off, where MeJA treatment significantly enhanced saponin accumulation while suppressing biomass growth, exemplifies the classic “growth-defense trade-off” in plants. Recent studies on *Panax notoginseng* provide molecular insights into this phenomenon. Research has shown that MeJA acts as a potent elicitor that upregulates key genes involved in the terpenoid backbone and saponin biosynthesis pathways, effectively redirecting metabolic flux from primary growth towards the production of defensive secondary metabolites like saponins. This mechanistic understanding aligns with our findings, where the application of MeJA likely triggered a similar transcriptional reprogramming, prioritizing the synthesis of valuable saponins over biomass accumulation. Furthermore, the role of jasmonates in coordinating defense responses, including the regulation of root system architecture under stress, supports the view that the biomass stagnation observed in our adventitious roots is an integral part of a coordinated, MeJA-triggered defense strategy. Therefore, the use of MeJA is an effective strategy to enhance the pharmaceutical value of *P. notoginseng* adventitious root cultures by boosting their saponin content, despite the cost to growth.

### 2.5. Metabolomic Analysis of Methyl Jasmonate Induction

#### 2.5.1. Identification of Saponin Monomers

During the identification of ginsenoside and notoginsenoside monomers, more MS^2^ fragment ions were detected in negative ion mode, making it more suitable for qualitative analysis. The tetracyclic structure of ginsenoside aglycones is stable and difficult to cleave, while the sugar moieties attached to the core are easily cleaved. In negative ion mode, the characteristic fragment ions of protopanaxadiol (PPD)-type aglycones are m/z 459, and those of protopanaxatriol (PPT)-type aglycones are m/z 475. The mass spectrometry fragmentation patterns of saponins generally follow this rule, and by comparing fragment ion information, triterpenoid saponins can be identified [22], especially for distinguishing isomers.

Notoginsenoside R1 is a unique saponin monomer in *P. notoginseng*, with the molecular formula C_47_H_80_O_18_, retention time (RT) 9.57 min, and a quasi-molecular ion peak at 931.5219 [M-H]^−^. The characteristic MS^2^ fragment ions were formed by the loss of one glucose (Glc) to produce 774.1094 [M-H-Glc]^−^, the loss of one xylose (Xyl) and one Glc to produce 637.4309 [M-H-Xyl-Glc]^−^, and the loss of one Xyl and two Glc to produce 475.3781 [M-H-Xyl-Glc-Glc]^−^ (Supplementary Figure S2). Ginsenoside Rg1, a PPT-type saponin, has the molecular formula C_42_H_72_O_14_, RT 9.89 min, and a quasi-molecular ion peak at 799.4823 [M-H]^−^, with an additional 45 Da formate ion forming a quasi-molecular ion peak at 845.4881 [M+HCOO]^−^. The loss of one Glc produced 637.4296 [M-H-Glc]^−^ and 475.3780 [M-H-Glc-Glc]^−^ fragment ions. Ginsenoside Rb1, a PPD-type saponin, has the molecular formula C_54_H_92_O_23_, RT 11.94 min, and a quasi-molecular ion peak at 1107.5911 [M-H]^−^. The loss of one Glc produced 945.5422 [M-H-Glc]^−^, 783.4874 [M-H-Glc-Glc]^−^, 765.4790 [M-H-Glc-Glc-H_2_O]^−^, 621.4358 [M-H-Glc-Glc-Glc]^−^, and the PPD characteristic ion 459.3807 [M-H-Glc-Glc-Glc-Glc]^−^.

Ginsenoside Re, a PPT-type saponin, has the molecular formula C_48_H_82_O_18_, RT 9.83 min, and is an isomer of the PPD-type ginsenoside Rd. The two were distinguished based on their MS^2^ fragment ions. Ginsenoside Re has a quasi-molecular ion peak at 945.5369 [M-H]^−^, with fragment ions formed by the loss of one rhamnose (Rha) to produce 799.4810 [M-H-Rha]^−^, the loss of one Glc to produce 783.4882 [M-H-Glc]^−^, the loss of one Rha and one Glc to produce 637.4309 [M-H-Rha-Glc]^−^, and the PPT characteristic ion 475.3779 [M-H-Rha-Glc-Glc]^−^. Ginsenoside Rd, a PPD-type saponin, has RT 12.52 min and forms quasi-molecular ion peaks at 945.5399 [M-H]^−^, 783.4881 [M-H-Glc]^−^, 765.4775 [M-H-Glc-H_2_O]^−^, 621.4354 [M-H-Glc-Glc]^−^, and the PPD characteristic ion 459.3832 [M-H-Glc-Glc-Glc]^−^.

**Figure 6:**
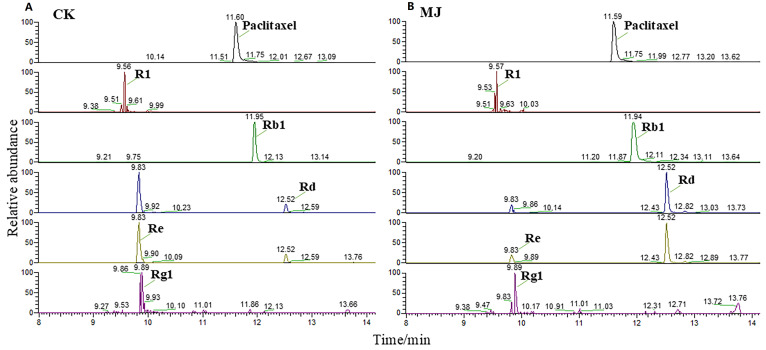
Chromatographic peaks of the main saponin monomers in *Panax notoginseng*. (**A**,**B**) are the chromatographic peaks of control group(CK) and 200 µM methyl jasmonate treatment groups, respectively. Paclitaxel internal standard chromatographic peak shape is good, the peak location is similar to the main saponin monomer, and can be separated. Ginsenosides Rd and Re were isomers. The peak height and peak area of Rd in methyl jasmonate treatment group were significantly higher than those in CK group.

A total of 4 notoginsenoside monomers and 10 common ginsenoside monomers were identified (Table 1). Based on the total saponin content in adventitious roots and the chromatographic peak areas of saponin monomers in LC-MS, MeJA was shown to be an effective elicitor for promoting the accumulation of total saponins in *P. notoginseng*. Additionally, MeJA exhibited heterogeneous induction effects on different saponin monomers. Rd (PPD) and Re (PPT) are isomers, and the chromatographic peak heights and areas of these isomers showed significant differences (Figure 6), indicating that MeJA favors the synthesis of PPD-type saponin monomers.

#### 2.5.2. Multivariate Statistical Analysis of Metabolites Induced by Methyl Jasmonate

##### Principal Component Analysis

**Figure 7:**
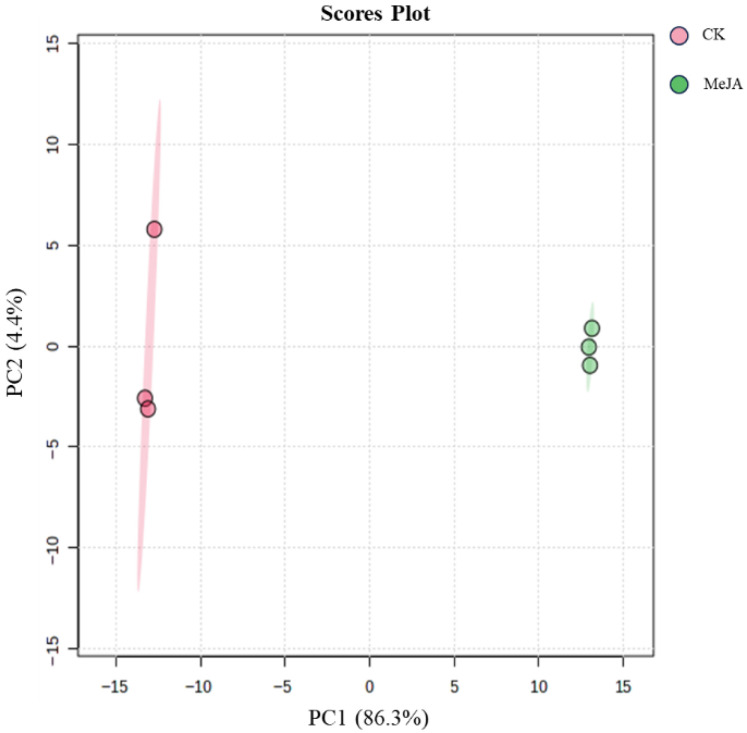
Principal component analysis scores of metabolites in *Panax notoginseng* adventitious roots induced by 200 µM methyl jasmonate.

After preprocessing the metabolomics data, the peak areas were log10-transformed, and principal component analysis (PCA) was performed. The PCA score plot is shown in Figure 7. The control (CK) and MeJA-treated groups were clearly separated. The first principal component (PC1) accounted for 86.3% of the variance, and in PC2, the MeJA group showed good clustering, while the CK group showed some dispersion. Overall, the two-component PCA model explained 90.7% of the variance, indicating that MeJA treatment significantly altered the metabolite profile of *P. notoginseng* adventitious roots.

##### Orthogonal Partial Least Squares-Discriminant Analysis

**Figure 8:**
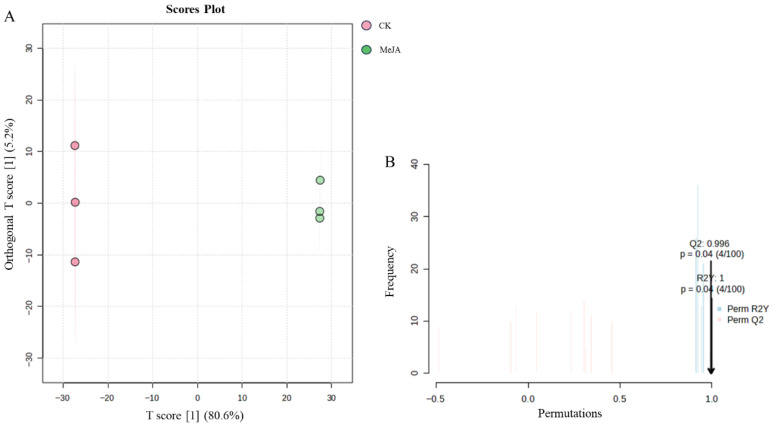
OPLS-DA analysis of metabolites in *Panax notoginseng* adventitious roots induced by methyl jasmonate. CK refers to the control group, and MeJA represents the treatment with 200 µM Methyl Jasmonate. (**A**) is orthogonal partial least squares discriminant analysis, (**B**) is permutation test.

Orthogonal partial least squares-discriminant analysis (OPLS-DA) was used to establish a model between metabolite expression levels and group classification. The OPLS-DA score plot (Figure 8A) showed clear separation between the two groups, with good clustering within each group. To avoid overfitting, the model was validated using the correlation coefficient R^2^ and the predictive ability Q^2^. The results showed that R^2^Y was 1, and Q^2^ reached 0.996. A permutation test (100 iterations) confirmed that the Q^2^ and R^2^Y values were significant (*p* < 0.05, Figure 8B), indicating that the model was accurate and not overfitted. This confirmed the reliability of using VIP > 1 to screen for differential metabolites.

#### 2.5.3. Screening of Differential Metabolites

**Figure 9:**
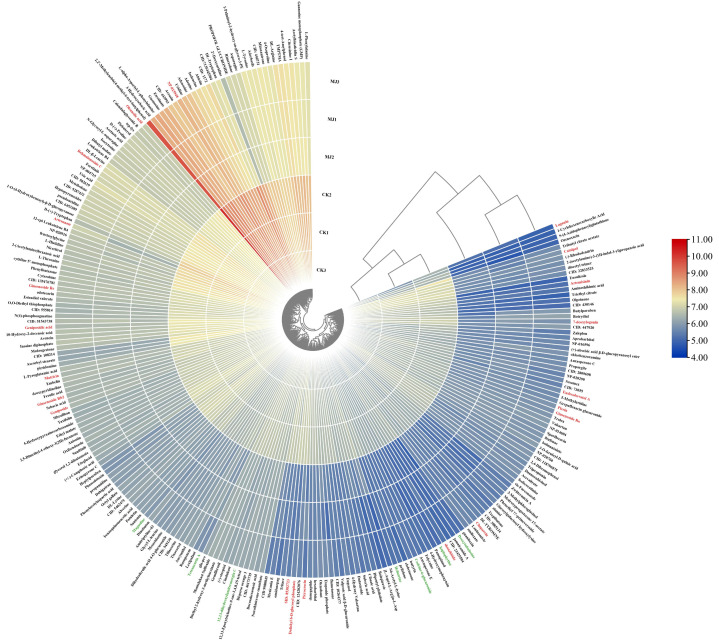
Heat map of differential metabolites in *Panax notoginseng* adventitious roots induced. The metabolites screened by *p* < 0.05 and |log2(FC)| > 1 were further screened with VIP > 1 as the threshold, and 242 differential metabolites were identified. Heat maps were drawn after the peak area was converted by log10. On the periphery of the heat map are metabolite names, red fonts are terpenoid, green fonts are alkaloid compounds; the starting plane and center of the circle on the right are cluster trees, which represent the similarity of clustering and metabolite expression patterns in different groups, respectively. It can be seen that samples of the two groups are clustered into a cluster, and the similarity of various metabolic compounds is good.

Under the set thresholds, a total of 328 differential metabolites were identified, of which 86 compounds could not be identified in multiple databases. The remaining 242 identified differential metabolites were log10-transformed, and a heatmap was used to visualize the differential metabolites (Figure 9). Under MeJA induction, the content of most differential metabolites in *P. notoginseng* adventitious roots was downregulated. The differential metabolites mainly consisted of terpenoids (e.g., Ginsenoside Re, Picein, Matricin, Eudesobovatol A, Chaparrin, Artesunate, Picrocrocin, etc., highlighted in red in Figure 9), alkaloids (e.g., Tetraneurin A, Magnoline, Heliotrine, Haplophytine, Deoxytubulosine, etc., highlighted in green in Figure 9), flavonoids (e.g., Taxifolin, Patuletin), and various fatty acids (Supplementary Table S2).

#### 2.5.4. KEGG Pathway Enrichment

**Figure 10:**
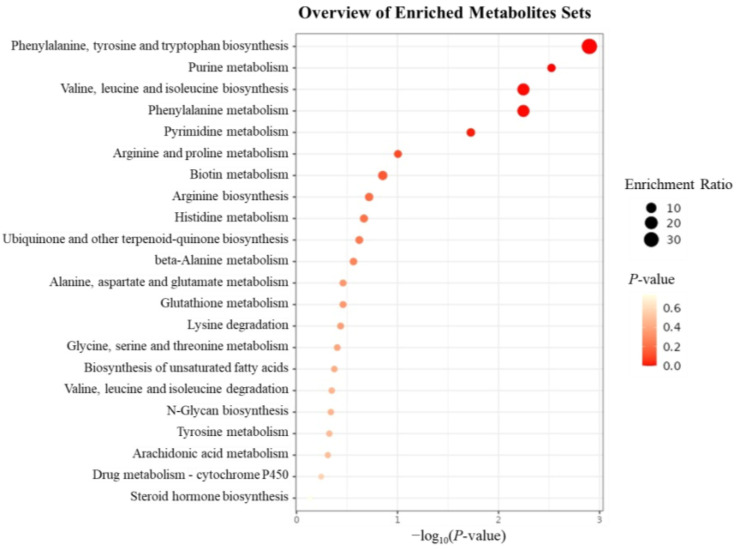
Bubble diagram of KEGG enrichment analysis of differential metabolites. The enrichment analysis was carried out on 242 differentiated metabolites that were screened and identified. The left side of the body was the enriched metabolic pathway, the size of the bubble represented the enrichment rate, and the color depth represented the *p*-value of significance.

The 242 identified differential metabolites were subjected to KEGG pathway enrichment analysis. As shown in Figure 10, 20 metabolic pathways were significantly enriched, including phenylalanine and tyrosine biosynthesis, purine metabolism, valine, leucine, and isoleucine biosynthesis, phenylalanine metabolism, pyrimidine metabolism, arginine and proline metabolism, biotin metabolism, arginine biosynthesis, histidine metabolism, ubiquinone and terpenoid-quinone biosynthesis, β-alanine metabolism, alanine, aspartate, and glutamate metabolism, glutathione metabolism, lysine degradation, glycine, serine, and threonine metabolism, unsaturated fatty acid biosynthesis, valine, leucine, and isoleucine degradation, N-glycan biosynthesis, tyrosine metabolism, and arachidonic acid metabolism. *P. notoginseng* is known to contain a variety of amino acids, and under MeJA induction, the chromatographic peak areas of many amino acids were significantly reduced. The KEGG enrichment results also indicated that MeJA primarily affected the synthesis and metabolism of various amino acids in *P. notoginseng* adventitious roots, reducing their biosynthesis rather than promoting it. The decrease in amino acids may indicate that MeJA redirects metabolic flux from primary metabolism towards the biosynthesis of secondary metabolites, such as saponins.

### 2.6. Effect of Methyl Jasmonate on Physiological Indicators of Adventitious Roots

**Figure 11:**
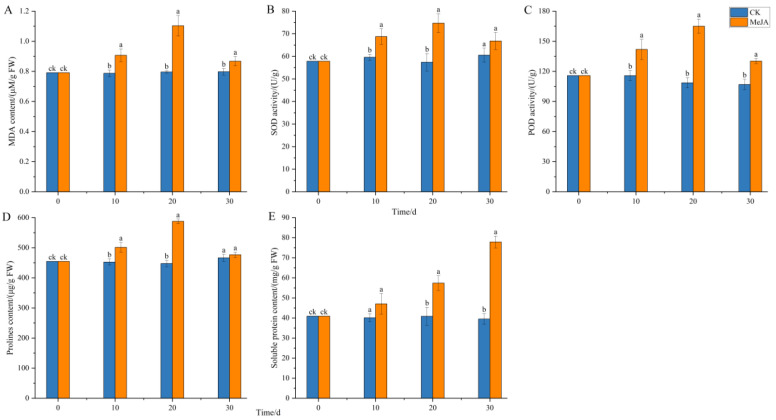
Effects of methyl jasmonate on physiological and biochemical indicators of *Panax notoginseng* adventitious roots. (**A**) represents the MDA content; (**B**) represents SOD activity; (**C**) represents POD activity; (**D**) is the PRO content; (**E**) represents the soluble protein content. CK refers to the control group, and MeJA represents the treatment with 200 µM Methyl Jasmonate. Different lowercase letters at the same time indicate *p* < 0.05. Error bars represent the standard error of each mean (n = 3).

As shown in Figure 11, 200 μM MeJA significantly increased the activities of SOD and POD enzymes, with enzyme activities showing an initial increase followed by a decrease over the cultivation period (Figure 11B,C). This trend was similar to the increase in fresh weight of adventitious roots, possibly due to the continuous increase in reactive oxygen species (ROS) exceeding the scavenging capacity of antioxidant enzymes, leading to some cellular damage and reduced growth. The MDA content also showed an initial increase followed by a decrease, reaching a peak of 1.1 μM/g FW at 20 days, significantly higher than the control group, and then decreasing slightly at 30 days but still significantly higher than the control (Figure 11A). Osmotic regulators such as proline (PRO) and soluble protein also accumulated under MeJA induction. The PRO content initially increased significantly and then decreased to a level similar to the control at 30 days, while the soluble protein content continued to accumulate over time, reaching 1.97 times that of the control group at 30 days. These results indicate that MeJA significantly increased the antioxidant enzyme activity and lipid peroxidation level in *P. notoginseng* adventitious roots, altering membrane permeability and cellular metabolism, and enhancing plant defense through the accumulation of osmotic regulators. MeJA may induce the accumulation of total saponins in *P. notoginseng* by triggering ROS bursts and activating plant defense responses.

### 2.7. Effect of Methyl Jasmonate on the Expression of Ginsenoside Biosynthesis Genes in P. notoginseng

**Figure 12:**
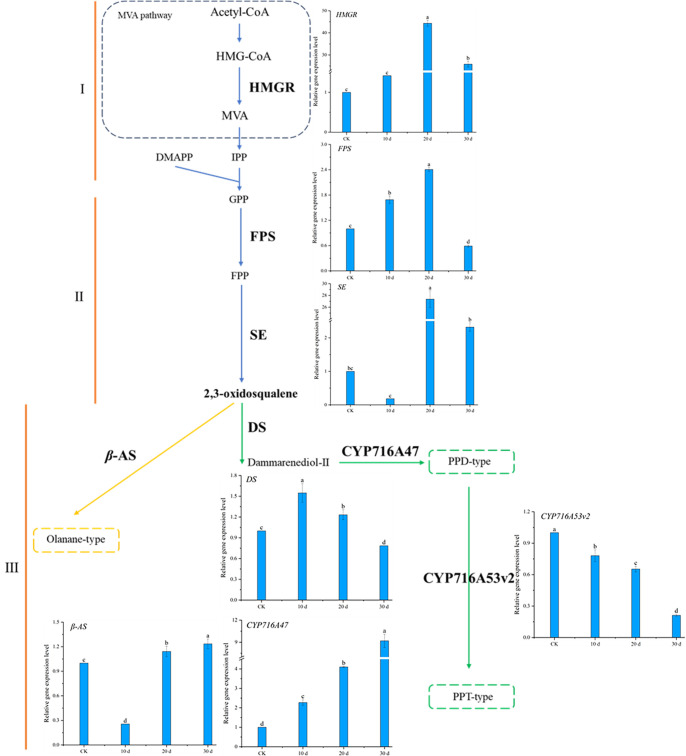
Ginsenoside synthesis pathway and related gene expression levels in *Panax notoginseng*. The epression levels of target genes were normalized in CK group. (**I**–**III**) represent the three stages of ginsenoside synthesis, respectively. Gene abbreviation: *HMGR*: 3-hydroxy-3-methylglutaryl CoA reductase; *FPS*: Farnesyl diphosphate synthetase; *SE*: Squalene epoxidase; *DS*: Damalenediol (**II**) synthetase; *β-AS*: *β*-Vanillin synthase; *CYP716A47*, *CYP716A53v2*: Cytochrome P450. Different lowercase letters indicate that the expression level of the gene at different times is *p* < 0.05.

RT-qPCR results (Figure 12) showed that the expression of *HMGR*, the first rate-limiting enzyme gene in the mevalonate (MVA) pathway, and *FPS*, a key branch point enzyme gene, gradually increased over the cultivation period, peaking at 20 days, after which *HMGR* and *FPS* expression decreased. Compared to the control, *HMGR* expression remained significantly upregulated at 30 days, while *FPS* expression was slightly downregulated. *SE*, a rate-limiting enzyme gene in triterpenoid and phytosterol biosynthesis, showed a similar expression pattern to *HMGR*. The expression patterns of *DS* and *β-AS*, the first key enzyme genes in the triterpenoid saponin biosynthesis pathway, which catalyze the synthesis of dammarane-type and oleanane-type ginsenosides from 2,3-oxidosqualene, differed significantly. *DS* expression initially increased significantly and then gradually decreased, while *β-AS* expression initially decreased significantly and then increased over the cultivation period (Figure 12III). *CYP716A47*, the enzyme gene catalyzing the formation of PPD-type saponin monomers from dammarenediol, showed gradually increasing expression over the cultivation period, with expression at 30 days being 9.2 times that of the control. In contrast, *CYP716A53v2*, the enzyme gene catalyzing the formation of PPT-type saponin monomers from PPD, showed downregulated expression from 10~30 days, with expression at 30 days being only 1/5 that of the control.

These results indicate that under MeJA induction, the expression of *HMGR*, *FPS*, *SE*, *DS*, and *CYP716A47* genes in the ginsenoside biosynthesis pathway was significantly upregulated, promoting the synthesis of ginsenosides in *P. notoginseng* adventitious roots. The downregulation of *CYP716A53v2* expression favored the synthesis and accumulation of PPD-type saponin monomers, consistent with the LC-MS results showing larger chromatographic peak areas for PPD-type saponin monomers compared to PPT-type monomers.

The gene expression patterns elucidated in this study provide a mechanistic breakthrough in understanding how methyl jasmonate (MeJA) fine-tunes the ginsenoside profile in *P. notoginseng*. It is well established in the literature that MeJA acts as a potent elicitor, broadly upregulating the early steps of the terpenoid backbone pathway (the MVA pathway) in *Panax* species. This is consistently demonstrated by the induction of genes such as *HMGR*, *FPS*, and *SE*, a pattern that our results firmly corroborate. However, the prevailing model of MeJA as a general amplifier of the pathway fails to explain the specific compositional shifts in the final saponin mixture.

The key innovation of our work lies in deciphering the precise regulatory logic at the critical branch point. We demonstrate for the first time in *P. notoginseng* adventitious roots that MeJA orchestrates a differential transcriptional program at the level of the downstream cytochrome P450 enzymes. The concurrent upregulation of *CYP716A47* (a PPD-type synthase) and downregulation of *CYP716A53v2* (a PPT-type synthase) represents a novel finding. This opposing regulation provides the first definitive genetic explanation for the preferential accumulation of PPD-type ginsenosides. It moves beyond the previously reported concurrent upregulation of both P450s by other jasmonic acid derivatives [23], and crucially, it also contrasts with the natural expression pattern where CYP716A53v2 is highly expressed in high-saponin roots [24]. Our demonstration that MeJA suppresses CYP716A53v2 not only perfectly accounts for the metabolic shift in our data but also reveals its power in redirecting inherent metabolic tendencies, thereby providing a key insight for the directed metabolic engineering of ginsenoside profiles.

Therefore, our study moves beyond the established paradigm. We reposition MeJA from a non-specific stimulant to a discriminatory regulator capable of actively redirecting metabolic flux. This insight is pivotal for metabolic engineering, as it identifies a strategic leverage point for tailoring adventitious root cultures to enrich for therapeutically preferred PPD-type saponins, thereby significantly enhancing the biotechnological value of our established culture system.

## 3. Materials and Methods

### 3.1. Induction and Proliferation of Callus

Plant materials (seeds and seedlings) of *Panax notoginseng* (Burk.) F.H. Chen were obtained from Wenshan, Yunnan Province, China. The identity was confirmed by Prof. Wenlan Li (College of Life Science and Technology, Guangxi University). Roots, stems, and leaves of one-year-old *P. notoginseng* were disinfected and inoculated into callus induction medium (Supplementary Table S1). Explants (roots, stems, and leaves) of *Panax notoginseng* seedlings were first washed with detergent for 10 min and thoroughly rinsed under running tap water for over 30 min. After being transferred into a laminar flow hood, they were surface-sterilized with 75% ethanol for 30 s, followed by two rinses with sterile distilled water. Subsequently, the explants were treated with either 0.1% (*w*/*v*) HgCl_2_ or a combination of 0.1% (*w*/*v*) HgCl_2_ and 1% (*v*/*v*) sodium hypochlorite (NaClO). Finally, they were rinsed 5–6 times with sterile distilled water, blot-dried on sterile filter paper, and inoculated onto solid MS medium (supplemented with 30 g/L sucrose and solidified with 0.7% agar, pH 5.8–6.0). The cultures were maintained at 25 ± 2 °C in the dark for 30 days, and the callus induction rates of different explants were recorded.

### 3.2. Adventitious Root Differentiation

Based on preliminary experiments, the adventitious root differentiation medium was determined to be 1/2 MS + IBA 2.0 mg/L + NAA 2.0 mg/L (supplemented with 30 g/L sucrose and solidified with 0.7% agar, pH 5.8–6.0). In this experiment, callus derived from leaf explants, which had been subcultured four times, was used. Callus and leaves of *P. notoginseng* were inoculated into the differentiation medium and cultured at 25 ± 2 °C in the dark. The differentiation and growth of adventitious roots were recorded at 30 and 50 days.

### 3.3. Inoculation Amount and Sucrose Concentration in the Medium

The effects of different inoculation amounts (2, 3, 4 g/L) and sucrose concentrations (20, 30, 40, 50 g/L) on the biomass and total saponin content of adventitious roots were investigated. Adventitious roots differentiated from leaves were gently separated from the callus and weighed to the target inoculation amount (±0.01 g/L) before being transferred to liquid medium. The cultures were maintained at 25 °C, 120 rpm in the dark for 30 days, after which biomass and total saponin content were measured.

### 3.4. Effect of Methyl Jasmonate on Adventitious Root Growth and Total Ginsenoside Content

The concentration of MeJA (200 μM) used in this study was selected based on preliminary screening experiments which evaluated its effects at various concentrations. Methyl jasmonate was sterilized by filtration through a 0.22 μm membrane. 200 μM MeJA was added to the liquid medium (1/2 MS + IBA 2.0 mg/L + NAA 2.0 mg/L + 5% sucrose). MeJA was added to the suspension culture medium at the time of inoculation (day 0). The control group received an equivalent volume of sterile water. Samples were harvested at 0, 10, 20, and 30 days post-inoculation for analysis. Statistical analysis showed no significant difference between the two controls, so sterile water was used as the control in subsequent experiments. Each flask was inoculated with 3 g/L of adventitious roots and cultured at 25 °C, 120 rpm in the dark for 30 days. Samples were taken every 10 days to measure biomass and total saponin content. Samples were also collected, flash-frozen in liquid nitrogen, and stored at −80 °C for physiological and biochemical analysis and RNA extraction.

### 3.5. Biomass and Total Saponin Content Measurement

(1) Fresh weight: Harvested adventitious roots were filtered through a 100-mesh sieve to remove the culture medium, washed three times with deionized water, and dried with filter paper before weighing to obtain fresh weight.
(2) Dry weight: The fresh weight of adventitious roots was dried in a 55 °C oven to a constant weight (approximately 2 days) and then weighed to obtain dry weight.
(3) Total saponin content: Dried adventitious roots were ground into powder and extracted twice with 12 volumes of water-saturated n-butanol using ultrasonic assistance at 40 °C for 50 min [25]. After evaporating the n-butanol, the residue was dissolved in methanol, and the total saponin content was determined using the sulfuric acid-vanillin colorimetric method [26].
(4) Standard curve: 3.1 mg of ginsenoside Re standard was accurately weighed and dissolved in methanol to a final volume of 10 mL, resulting in a standard solution concentration of 0.31 mg/mL. A 10 mL centrifuge tube was placed on ice, and 0.1, 0.2, 0.3, 0.4, and 0.5 mL of the standard solution were added to the tube, followed by 0.4, 0.3, 0.2, 0.1, and 0 mL of methanol to bring the total volume to 0.5 mL. A blank control was prepared using 0.5 mL of methanol. Then, 5 mL of 72% sulfuric acid and 0.5 mL of 8% vanillin in ethanol were added, mixed well, and incubated in a 60 °C water bath for 10 min. After the water bath, the reaction was immediately stopped by placing the tube in ice water for 15 min. Absorbance was measured at 544 nm. A standard curve was plotted with absorbance (y) against ginsenoside Re concentration (x), yielding the regression equation y = 0.7222x + 0.0901, with an R^2^ value of 0.9996.

### 3.6. LC-MS Conditions

The sample solution obtained from 3.5(1) was filtered through a 0.22 μm organic membrane before injection. For LC-MS analysis, 0.1 mg/mL paclitaxel was added as an internal standard. The chromatographic column was an ACQUITY UPLC BEH C18 column (2.1 × 100 mm, 1.7 µm; Waters, Milford, MA, USA), with a column temperature of 25 °C. The mobile phase consisted of (A) water and (B) methanol. The solvent gradient was as follows: 0~1 min, 25~33% B; 1~5 min, 33~33% B; 5~7 min, 33~41% B; 7~9 min, 41~41% B; 9~13 min, 41~59% B; 13~19 min, 59~59% B. The flow rate was 0.3 mL/min, and the injection volume was 5 µL. The mass spectrometer was a Thermo Scientific Q Exactive quadrupole-orbitrap high-resolution mass spectrometer, with the following conditions: ESI ion source, Full MS/dd-MS^2^ acquisition mode, positive and negative ion modes, positive ion spray voltage 3500 V, negative ion 3300 V, capillary temperature 320 °C, auxiliary gas temperature 350 °C, flow rate 10 L/min, sheath gas flow rate 35 L/min, mass scan range m/z 100~1500.

### 3.7. Effect of Methyl Jasmonate on Physiological Indicators of Adventitious Roots

Fresh samples were ground into powder in liquid nitrogen, and 0.1 g was weighed and mixed with 2 mL of 50 mM phosphate buffer (PBS, pH 7.8). The mixture was centrifuged at 13,524× *g* for 5 min, and the supernatant was collected as the crude enzyme extract and kept on ice. The activities of antioxidant enzymes (SOD, POD) and the contents of MDA, soluble protein, and free proline were measured as described in the literature [27].

### 3.8. Real-Time Quantitative PCR

RNA was extracted using a manual method, and its concentration was measured. The integrity of the RNA was checked using 1% agarose gel electrophoresis. First-strand cDNA was synthesized using the SparkJade SPARKscript II All-in-one RT SuperMix for qPCR (With gDNA Eraser) kit (Sparkjade, China), with 1 µg of total mRNA used for reverse transcription. RT-qPCR was performed using the SparkJade 2 × SYBR Green qPCR Mix (With ROX) kit (Sparkjade, China), with 5-fold diluted cDNA as the template. Primers (Table 2) were designed using Primer 5 software, verified by NCBI, and synthesized by GenScript Biotech (Nanjing, China). The reaction program was as follows: 95 °C pre-denaturation for 30 s, followed by 40 cycles of 95 °C for 10 s and 60 °C for 30 s. A melting curve program was then performed: 95 °C for 15 s, 60 °C for 60 s, and 95 °C for 15 s. *GADPH* was used as the internal reference, and the relative gene expression levels were calculated using the 2^−ΔΔCt^ method [28]. All experiments were performed in triplicate.

### 3.9. Data Analysis

(1) Data were analyzed using SPSS Statistics 26 software. Differences between groups were determined by comparing the means of different data sets (*p*-value), with *p* < 0.05 indicating a significant difference, denoted by different lowercase letters. Additionally, the scale bars in the figures represent 1 cm.
(2) The raw LC-MS data were processed using Compound Discoverer 3.0 software for untargeted metabolite analysis. ChemSpider and mzCloud databases were used to match molecular formulas, exact molecular weights, and MS^1^ and MS^2^ spectra for metabolite identification. Saponin monomers were further characterized using Thermo Xcalibur 4.1 software, with all identified notoginsenosides and ginsenosides having a mass error within 10 ppm. LC-MS data were preprocessed, and peak areas were log10-transformed for multivariate statistical analysis. Principal component analysis (PCA) was used to obtain an overview of sample distribution and identify potential outliers. Orthogonal partial least squares-discriminant analysis (OPLS-DA) was used to identify metabolites significantly contributing to clustering and discrimination. To reduce false positives, *p*-values were corrected using the false discovery rate (FDR). Metabolites with *p* < 0.05, |log2(FC)| > 1, and VIP ≥ 1 were considered differentially expressed. KEGG pathway enrichment analysis was performed.

## 4. Conclusions

This study established and optimized an adventitious root suspension culture system for *P. notoginseng* using tissue culture techniques. Compared to the control, the total saponin content in adventitious roots reached 47.44 mg/g DW, 2.34 times that of the control. Through metabolomics analysis, physiological indicator measurements, and the expression of ginsenoside biosynthesis genes, it was found that MeJA induced plant defense responses and upregulated the expression of ginsenoside biosynthesis genes, thereby increasing the total saponin content in *P. notoginseng* adventitious roots, particularly the synthesis of PPD-type saponin monomers. However, MeJA did not promote the synthesis of many metabolites and significantly reduced the synthesis of various amino acids. This study analyzed the induction effect of MeJA on *P. notoginseng* adventitious roots from a metabolic perspective, identifying a series of related metabolites and differential metabolites, which helps elucidate the biosynthesis mechanisms of ginsenosides and other differential metabolites and the induction mechanism of MeJA. The high controllability of plant cell suspension culture allows for the deliberate manipulation of the heterogeneity of secondary metabolites, maximizing the synthesis of desired secondary metabolites.

This study successfully transitions from methodological establishment to mechanistic discovery, offering a multi-faceted innovation in the field of *Panax notoginseng* biotechnology.

Firstly, from a technical standpoint, we have established a robust and practical platform for the sustainable production of *P. notoginseng* metabolites. The systematic optimization from explant selection to suspension culture conditions culminates in a protocol where leaf explants are unequivocally recommended due to their high induction rate, low contamination, and sustainable sourcing. This resolves critical practical bottlenecks for the industrial application of plant in vitro cultures.

Secondly, and most significantly, our research provides a paradigm shift in understanding the role of methyl jasmonate (MeJA). We move beyond its conventional characterization as a general elicitor to unveil its function as a “precision metabolic switch.” The core mechanistic innovation lies in our discovery of the differential transcriptional regulation at the pivotal branch point of the ginsenoside pathway. The coordinated upregulation of *CYP716A47* and downregulation of *CYP716A53v2* provides an elegant genetic explanation for the selective enrichment of PPD-type saponins, a finding that had remained elusive in previous studies. This offers a specific molecular target for future metabolic engineering efforts aimed at tailoring saponin profiles.

Finally, the integration of physiological, metabolomic, and transcriptional data provides a holistic model for elicitor action. We connect the initial MeJA-induced oxidative burst to the subsequent transcriptional reprogramming, which ultimately leads to a global reallocation of resources from primary metabolism (e.g., amino acids) towards the targeted synthesis of valuable secondary compounds.

In conclusion, this work is not merely a protocol for inducing saponin accumulation. It is a comprehensive study that establishes a scalable production system, deciphers its underlying regulatory mechanism, and provides a strategic blueprint for the precise engineering of plant specialized metabolism. The insights gained here are instrumental for advancing the bioproduction of high-value *P. notoginseng* saponins and have broad implications for the manipulation of secondary metabolism in other medicinal plants.

## Figures and Tables

**Table 1:** Common saponin structures identified in the extract of *Panax notoginseng*.

Type	Name	RT /min	Theoretical Molecular Weight [M-H]^−^	Measured Molecular Weight	Mass Error/ppm	MS^2^
	Notoginsenoside R1	9.57	931.5271	931.5219	−5.582	637.4309, 619.4176, 475.3781
	Notoginsenoside R2	11.11	767.4743	767.4709	−4.430	637.4300, 475.3779,
	Notoginsenoside Fa	11.75	1239.6379	1239.6330	−3.953	1239.6363, 1107.5931, 1077.5827, 945.5407, 783.4886, 459.3831
	Notoginsenoside Fc	12.06	1209.6273	1209.6239	−2.811	1209.6193, 1077.8514, 915.5327, 783.4890, 621.4357
PPD	Ginsenoside Rb1	11.94	1107.5956	1107.5911	−4.063	945.5422, 783.4874, 621.4358, 459.3807
PPD	Ginsenoside Rd	12.52	945.5428	945.5388	−4.230	945.5399, 783.4881, 621.4354, 459.3832
PPT	Ginsenoside Re	9.83	945.5428	945.5364	−6.769	945.5369, 799.4810, 783.4882, 637.4309, 475.3779
PPT	Ginsenoside Rg1	9.89	799.4849	799.4823	−3.252	637.4296, 475.3780
PPT	Ginsenoside Rg2	11.42	783.49	783.4860	−5.105	783.4895, 637.4303, 619.4180, 475.3782
PPT	Ginsenoside Ro	11.91	955.4908	955.4884	−2.512	955.4882, 793.4371, 569.3833
PPD	Ginsenoside Rc	12.2	1077.585	1077.5819	−2.877	1077.5860, 945.5431, 783.4896, 765.4788, 621.4369, 459.3830
PPD	Ginsenoside Rg3	13.44	783.49	783.4860	−5.105	783.4851, 621.4358, 459.3837
PPD	Ginsenoside Rb2	12.24	1077.585	1077.5845	−0.464	1077.5843, 945.5335, 783.4890, 621.4356, 459.3837
PPD	Ginsenoside Rb3	12.35	1077.585	1077.5817	−3.062	945.5474, 785.4865, 623.4396, 459.3848

**Table 2:** Genes and primers for ginsenoside biosynthesis (5′-3′).

Gene	Accession Number	Forward Primer	Reverse Primer
*GADPH*	KF815711	GATTCGGCATTGTTGAGG	CAGTGGGAACTCGGAAGG
*HMGR*	KJ804166	CCTGATAGCTGGGACATTC	CCGCAACTACTGCGTTAA
*FPS*	KJ804175	TGGGAAGATTGGCACAGA	TCGGCAAATACATCCTGAA
*SE*	KJ804171	TTTTGGATATGCCCTTTAC	CTTTCTCCCTCATTCGTT
*DS*	KJ804174	ATGTGGAAGCTGAAGGTTGCT	TTAAATTTTGAGCTGCTGGTGC
*β-AS*	KJ804177	AGGTAGGAGATGACGAGGTA	GCTGGGAACACTGTATCAA
*CYP716A47*	OR514680.1	ATGTCGTGTCGGGTGTTT	TTGGGACGCTTGCTTATT
*CYP716A53v2*	MZ277754.1	TTTCTGCGGTGCCTCGG	CTTGTGGATTGCTTCGGGTT

## Data Availability

Data are contained within the article and Supplementary Materials.

## Supplementary Materials

The following supporting information can be downloaded at: https://www.mdpi.com/article/10.3390/plants14223462/s1, Table S1. Callus induction rates of different hormone combinations. Table S2. Differential metabolites of *Panax notoginseng* adventitious roots induced by methyl jasmonate. Figure S1. Standard curve of total saponins in *Panax notoginseng*. Figure S2. Secondary mass spectrum characterization of Notoginseng saponins fragment ions.
